# Supplementary material for: A Hybrid Machine Learning Approach to Screen Optimal Predictors for the Classification of Primary Breast Tumors from Gene Expression Microarray Data
Source: Diagnostics (Basel). 2023 Feb 13;13(4):708. doi: 10.3390/diagnostics13040708 (PMC9955903; doi:10.3390/diagnostics13040708)
Supplement: Supplementary file 1 [file diagnostics-13-00708-s001.zip › diagnostics-2175839-supplementary.pdf]

Supplementary material

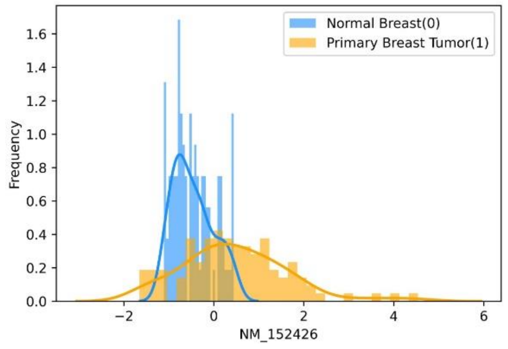

(a)

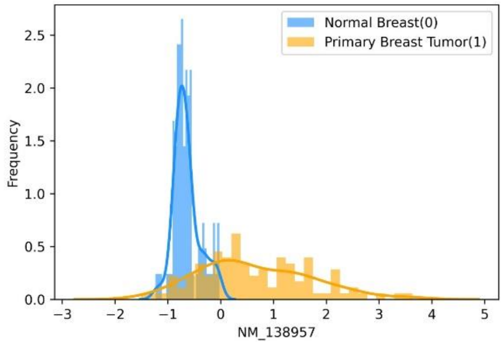

(b)

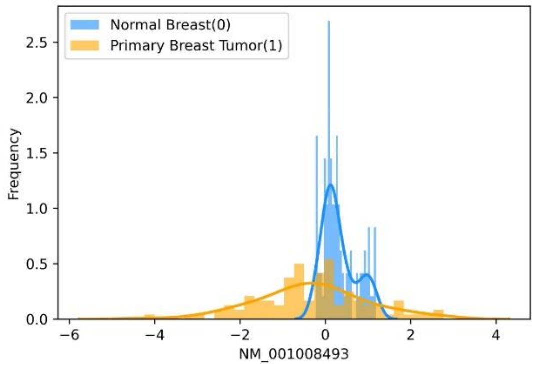

(c)

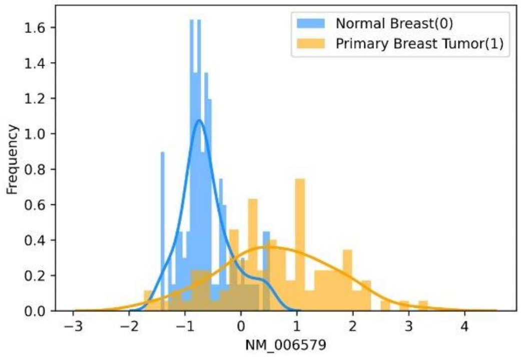

(d)

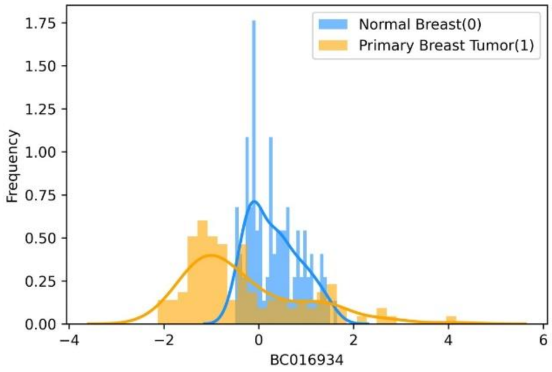

(e)

Supplementary Figure S1 (a-e). A pictorial representation of the histogram-based frequency distribution of the three optimal gene biomarkers (**a**) 'NM_152426', (**b**) 'NM_138957', (**c**) 'NM_001008493', (d) 'NM_006579', and (e) 'NM_BC016934', between the two classes of population (normal breast and primary breast tumor samples).
